# Supplementary material for: Pooled optical screening in bacteria using chromosomally expressed barcodes
Source: Commun Biol. 2025 Jun 3;8:851. doi: 10.1038/s42003-025-08268-5 (PMC12134211; doi:10.1038/s42003-025-08268-5)
Supplement: Supplementary file 2 — Supplemental Information [file 42003_2025_8268_MOESM2_ESM.pdf]

Supplementary Information for

Pooled optical screening in bacteria using chromosomally expressed barcodes

Ruben R. G. Soares[2*], Daniela A. García-Soriano[1*], Jimmy Larsson[1*], David Fange[1*#], Dvir Schirman[1], Marco Grillo[2], Anna Knöppel[1], Beer Chakra Sen[1], Fabian Svahn[1], Spartak Zikrin[1], Michael Ratz[3], Mats Nilsson[2#] & Johan Elf[1#]

1. Department of Cell and Molecular Biology, Uppsala University, SciLifeLab Uppsala, Sweden
2. Department of Biochemistry and Biophysics, Stockholm University, SciLifeLab Stockholm, Sweden
3. Department of Cell and Molecular Biology, Karolinska Institute, Stockholm, Sweden
* Equal contribution
[#] johan.elf@icm.uu.se, david.fange@icm.uu.se, mats.nilsson@scilifelab.se

Supplementary Figures

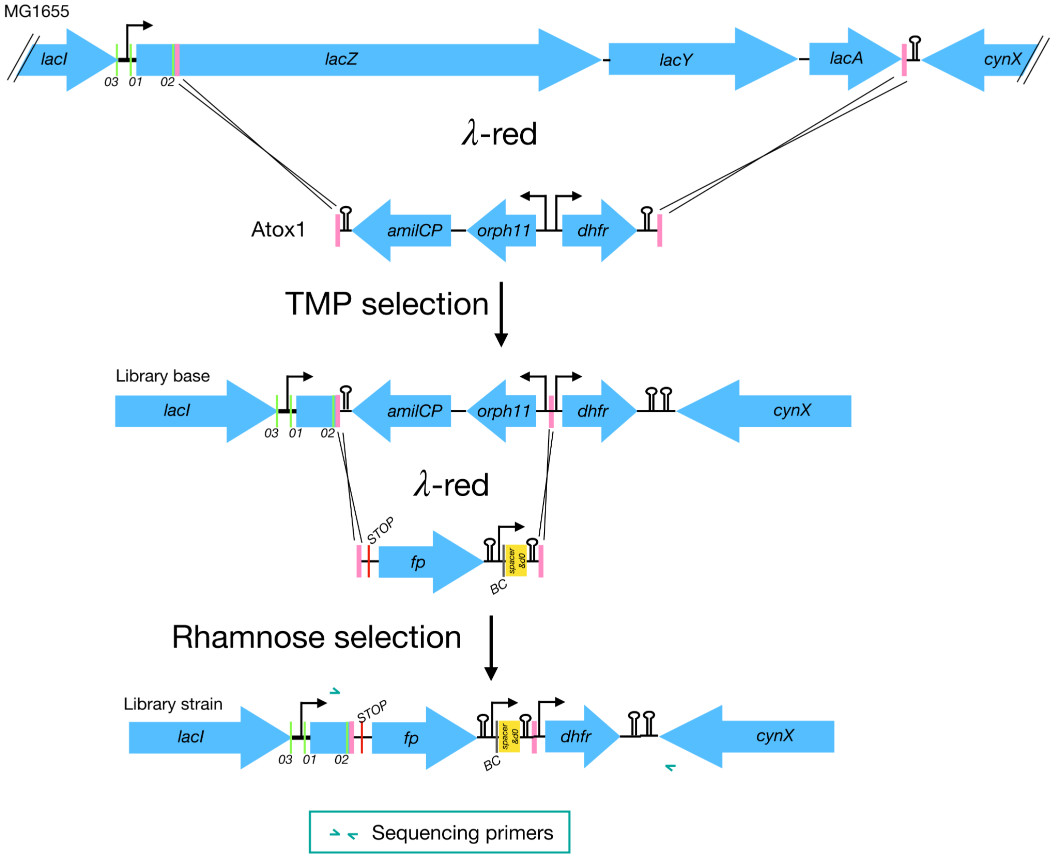

Supplementary Figure 1. Diagram of strain construction: The library base strain is generated by inserting the Atox1[19] cassette into the *lac*-operon of MG1655. All library strains, each with a different fp and BC, are generated from the library base strain. Primers for Sanger sequencing are found in the methods section.

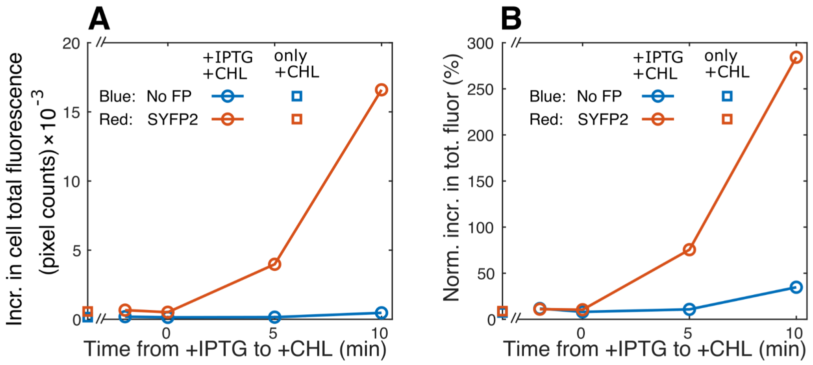

Supplementary Figure 2: Quantifying the timing of CHL action by inducing gene expression (+IPTG) and turning off protein synthesis (+CHL) at different time intervals. The lack of increase in total fluorescence when IPTG and CHL are added at the same time indicates that CHL stops protein synthesis within the time it takes to transcribe the mRNA encoding for SYFP2. (**A & B**) Increase in total cell fluorescence after various time of induction of gene expression (+IPTG) before adding chloramphenicol (+CHL) for cells either carrying an SYFP2 FP (red circles), or for cells without FP (blue circles). Cell fluorescence is measured at the time of +IPTG and then 30 min after +CHL to allow for FP maturation. For negative time cases (+CHL before +IPTG), fluorescence is measured at +IPTG and 30 min after +IPTG. Squares imply that no IPTG was added in the experiment. (A) Difference between after +CHL and before +IPTG in total cell fluorescence pixel counts. (B) Relative increase between +CHL and before +IPTG in percent.

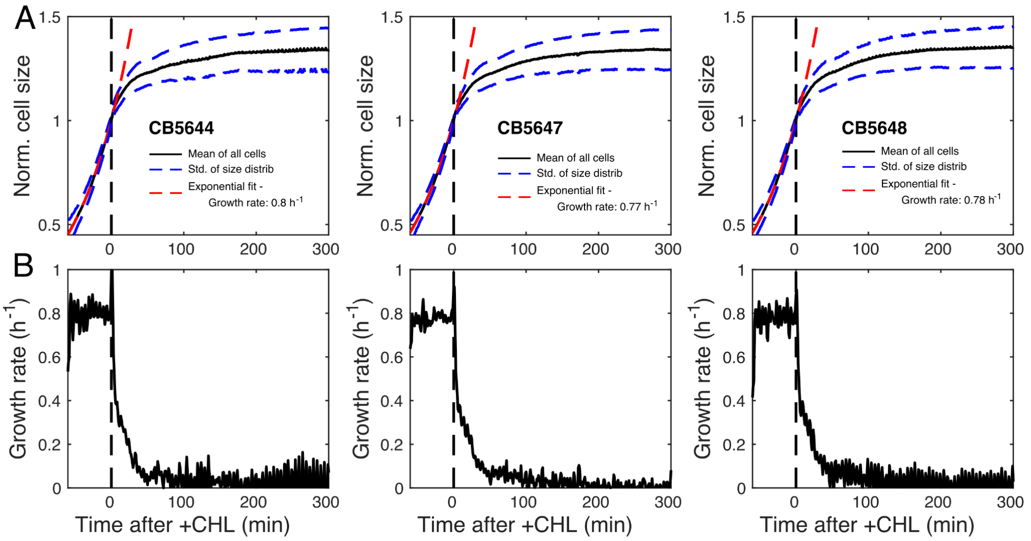

Supplementary Figure 3: Cell size and growth rate change as response to +CHL. (A) Cell size as function of time. Black solid line is the average of all cells in each experiment. Dash blue curves indicate one standard deviation for the distribution of cell sizes for each time point. Red dashed line shows regression to single exponential before swap to +CHL. Fitted growth rates before swap to +CHL are shown in insets. (**B**) Growth rate as function of time for three replicate experiments in (A). Black solid line is the average of all cells in each experiment.

Supplementary Tables

**Motif**	**DNA sequence**
R-homology arm	AATCCGCCGTTTGTTCCCACGGAGAATCCGACGGGTTGTTACTCGCTCACATTTAATGTT
FP-RBS	TTTGTTTAACTTTAAGAAGGAGA
terminator (L3S2P21)	CTCGGTACCAAATTCCAGAAAAGAGGCCTCCCGAAAGGGGGGCCTTTTTTCGTTTTGGTCC
T7-promoter	TAATACGACTCACTATAGGGAGA
Spacer	GGTTGACCTTTGTACATTAATTTAA
d0	GGGAGGACTCCCACAGTCACTGGGGAGTCCTCGAATACGAGCTGGGCACAGAAGATATGGCTT CGTGCCCAGGAAGTGTTCGCACTTCTCTCGTATTCGATTCCC
T7 terminator	CTAGCATAACCCCTTGGGGCCTCTAAACGGGTCTTGAGGGGTTTTTTG
L-homology arm	CATATCGAATTTACGGCTAGCTCAGTCCTAGGTATAGTGCTAGCGCAAGGAGACAAGAGA

Supplementary Table 1: DNA sequences of common motifs

		CB5644		CB5647		CB5648	
**BC**	**FP**	**median (min)**	**nr of lineages**	**median (min)**	**nr of lineages**	**median (min)**	**nr of lineages**
275	AzaleaB5	>300	161	>300	65	>300	156
280	DsRed.M1					124	30
283	FusionRed	290	106	>300	61	>300	71
284	FusionRed-M	>300	128	>300	44	>300	103
285	FusionRed-MQV	184	87	199	44	201	132
301	pHuji	56	210	66	370	60	221
308	TagRFP-T	77	100	81	102	83	151
309	super-TagRFP	125	102	133	111	134	225
312	TagRFP675	74	40	79	69	76	72
317	cgfmKate2	46	87	49	60	51	95
327	mCherry2-L	29	74	34	36	34	77
328	mApple	58	59	57	39	58	71
330	mCardinal			149	39	150	31
331	mCarmine	297	29	>300	29		
335	mCherry	47	118	48	88	48	155
337	mCherry-XL	42	121	49	76	48	147
338	mCherry2	31	131	35	115	34	157
353	mGarnet2			>300	34		
356	mGinger2	142	34				
357	mGrape1	63	36	79	36	77	62
363	mKOκ	151	65	158	61	157	71
367	mKO2	180	202	206	211	200	162
369	mKate S158A	67	173	70	145	72	280
370	mKate S158C	86	165	91	140	88	203
371	mKate M41G S158C	72	159	76	159	74	144
372	mKate	129	131	141	128	134	223
373	mKate2	81	158	88	140	86	171
378	mKelly2	>300	107	>300	51	>300	104
382	mMaroon1	88	95	89	67	84	107
384	mNectarine			68	25		
385	mNeptune			>300	20		
388	mNeptune2.5	148	78	171	68	163	62
395	mNeptune684	94	10				
402	mPlum	282	100	>300	73	>300	122
403	mPlum-E16P	95	78	108	85	113	99
404	mRFP1	55	147	59	164	62	169
409	mRFP1-Q66C	163	155	161	161	154	215
417	mRFP1.1			68	45		
418	mRFP1.2	43	108	45	94	44	103
424	mRaspberry	>300	38	>300	18	>300	35
429	mRed7Q1S1	>300	122	>300	80	>300	142
436	mRuby	>300	97	>300	26	>300	54
438	mRuby3	>300	184	>300	165	>300	170
450	mScarlet	240	124	245	43	268	72
454	mScarlet-H			>300	27	>300	30
457	mScarlet-I	46	111	49	89	53	68
462	mStable			>300	27		
464	mStrawberry	217	115	>300	72	237	100
468	mTangerine	43	108	96	73	110	128
474	mScarlet3	157	68	189	58	178	62

Supplementary Table 2. Maturation time statistics. Only FPs with > 4 decoded traps in an experiment are included. The cases where the median maturation time is longer than the fluorescence image acquisition time after +CHL are denoted > 300 min.

	A. Cluster analysis of all FPs			B. Detected maturation times in traps decoded as “no FP” (BC303)		
**Experiment**	**Nr. of outlier traps**	**Tot. nr. of traps**	**Fraction**	**Nr. of outlier traps**	**Tot. nr. of BC303 traps**	**Fraction**
CB5644	21	624	0.033	7	58	0.121
CB5647	30 (11)	550 (494)	0.055 (0.022)	6	52	0.115
CB5648	3	670	0.004	0	62	0.000
Total	54	1844	0.029 (0.02)	13	172	0.076

Supplementary Table 3: Frequencies of cell traps containing cells with phenotypic outliers. (**A**) Outlier traps contain cells which are phenotypically deviating from the majority cluster for each FP in Fig. 3. In CB5647, BC301 (pHuji) has a deviating number of decoded traps compared to the two other repeats (Fig. S4) and the number of phenotypic outliers are also high in this experiment (Fig. 3). The values in parenthesis are CB5647 excluding BC301 (pHuji). (**B**) Outlier traps contain cells which have a strong enough fluorescence signal to have a maturation time estimate although being decoded as “no FP” (BC303). The high false positive rate for BC303 is inline with the observation of BC303 being observed more frequently in the *in situ* genotyping compared to what is expected from NGS-based amplicon sequencing (Fig. 2).

1. Lysozyme reaction mix	Stock Conc.	Final Conc.	Volume (uL)
Lysozyme (Thermo scientific)	50 mg/ml.	250 ug/ml	
PBS-Tween 0,01%			

2. BSA solution	Stock Conc.	Final Conc.	Volume (uL)
BSA (Thermo scientific)	10%	1%	
PBS-Tween 0,01%			

3. Zombie transcription mixture	Stock Conc.	Final Conc.	Volume (uL)
Transcription buffer (Thermo scientific)	5x	1x	16
NTPs	25 mM	2 mM	6.4
MgCl2	25 mM	6 mM	19.2
Tween 20 (Promega)	1% (v/v)	0.1% (v/v)	8
Glycerol	50%	5%	8
T7 RNA Polymerase (Thermo scientific)	20 U/µl	2 U/µl	8
Riboprotect (Qiagen gdansk)	40 U/µl	1 U/µl	2
H2O mQ	-	-	12.4

4. PLP Hybridization mixture	Stock Conc.	Final Conc.	Volume (uL)
20x SSC	20x	2x	5
Ethylene Carbonate (Merck)	100%	5%	2.5
MgCl2	50 mM	15 mM	15
Tween 20	1% (v/v)	0.1% (v/v)	5
Padlock probe library (each probe)	0.46 uM	198 nM	21.25
Riboprotect (Qiagen gdansk)	40 U/µl	1 U/µl	1.25

5. SplintR ligation mixture	Stock Conc.	Final Conc.	Volume (uL)
SplinR Buffer (NEB)	10x	1x	6
Glycerol	50%	5%	6
Tween 20	1% (v/v)	0.1% (v/v)	6
Riboprotect (Qiagen gdansk)	40 U/µl	1 U/µl	1.5

SplintR Ligase (NEB)	25 U/uL	0.5 U/uL	1.2
H2O mQ	-	-	39.3
**6. RCA primer hybridization mixture**	**Stock Conc.**	**Final Conc.**	**Volume (uL)**
20x SSC	20x	2x	5
Ethylene Carbonate (Merck)	100%	5%	2.5
MgCl2	50 mM	15 mM	15
Tween 20	1% (v/v)	0.1% (v/v)	5
Gycerol	50%	1.25%	1.25
RCA primer **GGCTCCACTAAATAGACGCA**	1 uM	0.1 uM	5
H2O mQ	-	-	6.25
**7. RCA mixture**	**Stock Conc.**	**Final Conc.**	**Volume (uL)**
dNTPs	2.5 mM	250 uM	9
phi 29 buffer †	10x	1 x	9
Glycerol	50%	5%	9
BSA	20 μg/μl	0.2 μg/μl	0.9
phi 29 polymerase *	10 U/uL	1 U/uL	9
H2O mQ	-	-	53.1
**8. Labeling mixture**	**Stock Conc.**	**Final Conc.**	**Volume (uL)**
Detection oligos (IDT)	1 μM each	100 nM	6
4x SSC 40% Formamide	2x	1x	30
L-probe pool	1 μM total	0.1 μM total	6
H2O mQ	-	-	18
**9. Probe stripping mixture**	**Stock Conc.**	**Final Conc.**	**Volume (uL)**
Formamide	100%	90%	900
PBS-Tween	1x	0.1x	100

Supplementary Table 4: Reaction mixes used for *in situ* genotyping. * Wild-type phi29 DNA polymerase was transformed into *E. coli* BL21 (DE3) T1R pRARE2 cells. The cells were cultivated in Terrific Broth (TB) medium. Protein expression was induced with IPTG and the protein was purified by immobilized metal-ion chromatography (IMAC), followed by size exclusion chromatography (SEC). † The phi29 reaction buffer at 10x concentration is: 500 mM Tris-HCl (pH 8.3), 100 mM $MgCl_2$ & 100 mM $(NH_4)_2SO_4$
