# Supplementary material for: Pooled optical screening in bacteria using chromosomally expressed barcodes
Source: Commun Biol. 2025 Jun 3;8:851. doi: 10.1038/s42003-025-08268-5 (PMC12134211; doi:10.1038/s42003-025-08268-5)
Supplement: Supplementary file 3 — Description of Additional Supplementary Files [file 42003_2025_8268_MOESM3_ESM.pdf]

Description of Additional Supplementary Files

File Name: Supplementary Data 1
Description: DNA sequences for complete chromosome insert for all strains. BC and FP sequences are indicated by upper case.

File Name: Supplementary Data 2
Description: Padlock probe sequences.

File name: Supplementary Data 3
Description: Detection oligos, genotyping codes and L-probe sequences.
